# Gender-Based Analysis of the Psychological Impact of the COVID-19 Pandemic on Healthcare Workers in Spain

**DOI:** 10.3389/fpsyt.2021.692215

**Published:** 2021-07-20

**Authors:** Mayte López-Atanes, José Ignacio Pijoán-Zubizarreta, Juan Pablo González-Briceño, Elena María Leonés-Gil, María Recio-Barbero, Ana González-Pinto, Rafael Segarra, Margarita Sáenz-Herrero

**Affiliations:** ^1^Department of Psychiatry, Cruces University Hospital, Barakaldo, Spain; ^2^Department of Neurosciences, University of the Basque Country (UPV/EHU), Leioa, Spain; ^3^Department of Epidemiology, Cruces University Hospital, Barakaldo, Spain; ^4^Biocruces Bizkaia Health Research Institute, Barakaldo, Spain; ^5^Centro de Investigación Biomédica en Red de Salud Mental, CIBERSAM, Madrid, Spain; ^6^Department of Psychiatry, Araba University Hospital, Vitoria-Gasteiz, Spain; ^7^Bioaraba Health Research Institute, Vitoria-Gasteiz, Spain

**Keywords:** coronavirus—COVID-19, mental health, gender analysis, COVID-19, women, gender

## Abstract

**Purpose:** This study aims to analyze from a gender perspective the psychological distress experienced by the medical workforce during the peak of the pandemic in Spain.

**Methods:** This is a single-center, observational analytic study. The study population comprised all associated health workers of the Cruces University Hospital, invited by email to participate in the survey. It consisted of a form covering demographic data, the general health questionnaire-28 (GHQ-28), and the perceived stress scale (PSS-14). We used multivariant regression analysis to check the effect of gender on the scores. We used gender analysis in both design and interpretation of data following SAGER guidelines.

**Results:** Females made 74.6% of our sample, but their proportion was higher in lower-paid positions such as nursery (89.9%) than in higher-paid ones. The percentage of women categorized as cases with the GHQ-28 was 78.4%, a proportion significantly higher than in the male population (61.3%, *p* < 0.001). The multivariant regression analysis showed that being women, working as orderly hospital porters, and having a past psychiatric history were risk factors for higher scores in both the GHQ-28 and PSS-14.

**Conclusion:** Women and those with lower-paid positions were at risk of higher psychological distress and worse quality of life within the medical workforce during the first wave of the pandemic. Gender analysis must be incorporated to analyze this fact better.

## Introduction

The COVID-19 pandemic emerged as a viral infection caused by the Severe Acute Respiratory Syndrome Coronavirus 2 (SARS-CoV-2). The outbreak started in China and brought catastrophic economic, social, and psychological consequences. For people working in the healthcare sector, the situation was particularly stressful because there were no data on transmission dynamics and evidence-based recommendations on the protective measures needed. Our study took place in the Cruces University hospital, a third-level hospital in the Basque Country, Spain, covering more than 370,000 people. A total of 365 COVID-19 patients were admitted during the study period in this Hospital. By the end of June 2020, a total of 20,006 cases had been registered in the Basque Country (1).

During the first wave of the pandemic, when our study took place, Spain ranked first in the World in healthcare infections (2). A report published by the European Center for Disease Prevention and Control (ECDC) in April 2020 underscored that 20% of registered coronavirus cases in Spain were healthcare workers that probably became infected due to a lack of protective measures. Although there is not enough data on the estimates of COVID-19 infections among healthcare workers during the first peak, one study found that 11.2% of hospital porters had laboratory evidence of current or past coronavirus infection (3). Therefore, the healthcare population was at high risk of COVID-19 disease in Spain.

### The Pandemic and the Medical Workforce

Healthcare workers in the frontline against COVID-19 were particularly vulnerable to psychosocial distress (4). The medical workforce comprises doctors, nurses, medical residents, and assistants, each with their function, perspective, and share in responsibility and exposure to the virus. Past studies show that during the SARS outbreak in 2003 (a coronavirus that causes atypical respiratory disease such as SARS-CoV-2), more than 10% of healthcare workers directly involved with patient care developed post-traumatic stress symptoms 1 year after the epidemic (5). Years after the outbreak, these individuals presented a higher prevalence of psychiatric comorbidities like depression, anxiety, and drug abuse (particularly alcohol) than those non-directly involved (4, 6); also, up to 57% of interviewed hospital porters reported psychological distress when asked about their perceived stress and general health status (7). Based on this current data, we presumed that healthcare workers involved in patient care during the COVID-19 pandemic could develop mental health consequences.

The evaluation of the impact on the workforce's mental health became a priority during the first wave, and studies are already published. Most of them found significant differences in stress and anxiety symptoms by gender and job category (8–17). Meta-analytic studies demonstrate that being a woman and lower-paid jobs, such as nursery, are risk factors for higher psychological distress during the pandemic (18–23). In Spain, data from the MIND/COVID project, a multiple-cohort study of the mental health impact of the first wave of the COVID-19 pandemic, revealed that almost half of healthcare workers met the criteria for common mental disorders and 1 in 7 for a disabling mental disorder (24). Interestingly, they also found that being a woman was a risk factor for any mental disorder but protective for a suicidal plan or attempt during the pandemic (24, 25).

### COVID-19 and Gender

Gender inequality remains an important issue in European Union public health policies. The COVID-19 crisis created an imminent challenge for the provision of equal rights for women and men. During the first wave of the pandemic, a significant number of reports, publications, and public policies focused on the necessity of addressing gender inequity and establishing measures to ensure equality between women and men (26–28).

The European Institute for gender equality describes the scopes in which the pandemic negatively affected women (29). These include worse global health, increased unpaid work, economic hardship, domestic violence, and the marginalization of populations such as sex workers. In the particular case of Spain, the mandatory lockdown carried a 48% increase of calls to the gender-based violence helpline (27), presumably due to victim isolation and barriers to help-seeking (30). After the lockdown, schools and daycare centers' closening derived in more time dedicated to housework for both women and men. However, the number was still higher for women: they invested 18.4 h per week compared to 12.5 h of men (29). In the UK, women spent more time in caretaking duties and were more likely to change employment schedules and experience psychological distress (31).

Women in the healthcare sector constitute a group of particular importance. These women face the new difficulties derived from the lockdown. Before the COVID-19 crisis, these women were already at risk of poor mental health due to sexual harassment (32, 33), lack of work-life balance (34) and inferior career prospects compared to their men peers (35). Women in their medical training suffered from higher levels of suicide and depression due to work-family conflicts (36). Due to labor segregation, most of the health care workers in the frontline against the virus were, in fact, women. According to the World Health Organization (WHO), up to 70% of total healthcare workers are women, and this number is even higher in some professions such as nursing (37), with an estimate of 83% in Spain (27). In the Basque Health system, the proportion is about 80% (38). Not only were women more exposed to the virus, but they also were subject to new inequalities. Firew et al. (39) showed that women were less likely to be tested for COVID-19 infection than men; still, research on this topic is scarce.

### Sex/Gender-Sensitive Research

Although research on the psychological impact of the pandemic in the medical workforce has been extensive, most studies fail to include a gender-sensitive perspective. Gender analysis refers to the inclusion of a gender perspective in all the research steps, from project design to data interpretation and discussion. Guidelines such as Sex and Gender Equity in Research (SAGER) provide recommendations on incorporating a gender-based analysis into the various steps of the research process (40). Some studies highlight the fact that gender influences the response to the pandemic in the general population (41–44) but fail to include psychosocial gender-related variables in the discussion. For example, they approach gender (which is by definition a cultural construction) from a biological perspective to justify the different incidence of anxiety, depression, stress, and trauma-related symptoms in women (45). If we focus on the medical population, current published work barely alludes to gender as a core variable influencing the results and fails to provide gender-disaggregated data.

This study describes the psychological distress experienced by healthcare workers during the COVID-19 pandemic, including a gender-sensitive analysis. We hypothesize that being female is a risk factor for higher distress during the first wave of the pandemic.

## Methods And Analysis

### Objectives

The study's main objective was to describe the psychological impact of a high-stress situation such as the COVID-19 pandemic in healthcare workers focusing on gender differences. We hypothesized that women experience a higher level of psychosocial stress than men and, therefore, lower health quality. The secondary objective was to detect risk factors for higher psychological distress within the medical workforce.

### Study Design

This is a single-center, observational, analytic, prospective study conducted within the Department of Psychiatry of the Cruces University Hospital in collaboration with the Epidemiology Unit of the Biocruces Research Center. The study consisted of an online questionnaire sent by email to all the workers of the Cruces University Hospital. The invitation email had an introduction letter with a short description of the study and a link to the online formulary in a Google Forms platform. It included a statement of consent, for which participation in the survey implied consent by the individuals. As the survey was anonymous, voluntary, and did not include traceable data of the subjects, the ethics committee did not ask for signed informed consent.

### Participants

The study population comprised all the current employees of the Cruces University Hospital that figured in the hospital porters email database. These include Medical Residents, Qualified Doctors, Nurses, Nurse Assistants, Orderly hospital porters, and non-medical workers such as administrative hospital porters. The email database excludes those outsourcing labor services such as cleaning, cooking, and ambulance services.

### Recruitment

Recruitment took place took place from May 1 to Jun 30, 2020. We sent an invitation to participate in the study by the institutional email in May and a reminder in June. We closed the access to the survey by Jun 30. We used convenience sampling, and all completed surveys were included in the analysis.

### Measurements

#### Sociodemographic Information

Participants reported their age (in years), gender, occupational status, past psychiatric history, contact with COVID-19 positive patients, and diagnosis of COVID-19 infection.

#### General Health Questionary (GHQ-28)

The General Health Questionnaire (GHQ) (46) is a self-administered screening device that assesses the respondent's perceived sensation of changes in well-being. Created by Goldberg in 1979, it included 60 items, but shorter versions of 30, 28, 20, and 12 items exist and also show good validity (47). It has been widely used to detect psychiatric disorders in the community and non-psychiatric health care settings (48). We used the Spanish version of the GHQ-28 scale translated by Lobo et al. (49) available at the Centro de Investigación Biomédica en Red (CIBER) of mental health website (50). In this abbreviated version of 28 items, the perceived health is analyzed through four subscales of seven questions: somatic symptoms, anxiety/insomnia, social dysfunction, and severe depression. Responses are rated on a four-point Likert scale (0 to 3), and the higher the scores, the worst is the quality of health. The Spanish version was already tested by previous studies and showed good internal consistency (Chronbach alfa between 0.76 and 0.97) (47, 49, 51). We scored the scale with a binary method were Not at all, and No more than usual score 0, and Rather more than usual and Much more than usual score 1. Using this method, any score above 4 indicates the presence of distress or “caseness” (52).

#### Perceived Stress Scale (PSS-14)

Cohen introduced the Perceived Stress Scale in 1983 (53) and nowadays represents one of the most used tools for measuring psychological distress. This scale evaluates the degree to which individuals believe their life has been unpredictable, uncontrollable, and overloaded during the previous month. Its original form consists of a self-reported questionnaire of 14 items, with seven positively stated items and seven negatively stated, rated on a five-point Likert scale. Scores for the 14-item scale range from 0 to 56. The Spanish version used in our study was translated and validated by Remor (54). It showed a good correlation with similar scales (54, 55) and reliability related to internal consistency, showing Cronbach alfa values between 0.81 and 0.86 (53, 54).

### Gender Analysis

We followed the SAGER guidelines for incorporating the gender perspective into research (40). We chose gender instead of sex as we worked with the individual self-report of their identity instead of the biological attributes of each participant. Descriptive data is segregated by gender. In the interpretation of findings, past research was examined for gender bias, and we considered both social, cultural, and biological factors in the discussion.

### Statistical Analysis

Statistical analysis was carried out using IBM® SPSS® Statistics version 25.0 (IBM GmbH, Ehningen, Germany). Categorical data were presented as frequencies and percentages, and continuous variables as mean and standard deviation. Kolmogorov–Smirnov test was used to determine the normal distribution of the scale results. As data were not normally distributed, the non-parametric Mann-Whitney U test and Kruskal-Wallis were applied to compare two or more groups, respectively. We conducted a Spearman's Rho correlation test between age and the PSS-14 score. A significance level was set at a = 0.05; therefore, a *p*-value < 0.05 was considered statistically significant. Continuous data were described by mean value, and categorical data were characterized by frequency. Multiple linear regression analysis was conducted to identify variables predictive of, or associated with, psychological stress in the early stages of the COVID-19 epidemic. The multiple linear regression analysis was constructed in a stepwise fashion with the following covariates: age, gender, job category, marital status, and psychiatric history.

### Ethical Considerations

The Ethics Committee of the Basque country (CEIm-E) approved the study on Apr 1, 2020 (ID: PI2020056, code psicovid19). All procedures contributing to this work comply with the ethical standards of the relevant national and institutional committees on human experimentation and with the Helsinki Declaration of 1975 (Fortress Amendment, Brazil, October 2013). The Ethics Committee approved all procedures involving human subjects.

## Results

### Demographic Characteristics

Demographic Characteristics are shown in Table 1. Six hundred seventy three healthcare workers completed the survey in our study, but six did not report their gender and were not included in the analysis. Most of them (514 [76.4%]), were women, married (360 [53.5%]) and caregivers (343 [51%]). Only 78 participants (11.6%) reported previous psychiatric history. Of the total respondents, 193 were qualified doctors (28.7%), and 187 (27.8%) were nurses, comprising most of the sample. Most of the participants (353 [52.5%]) reported having been in the frontline caring for confirmed or suspected COVID-19 patients. Still, only 66 (9.8%) declared to have tested positive for COVID-19 at the survey moment. The mean age was 43.2, (SD: 11.81).

**Table 1:** Socio-Demographic features.

		**Overall**		**Females**		**Males**		
**Variable**	**Categories**	**Observed; *n* (%)**	**Missing; *n* (%)**	**Observed; *n* (%)**	**Missing; *n* (%)**	**Observed; *n* (%)**	**Missing; *n* (%)**	***p*-value**
Duty work responsibilities		653 (97.9)	14 (2.1)	503 (97.9)	11 (2.1)	150 (98.0)	3 (2.0)	0.038
	Medicine specialties	128 (19.2)		89 (17.3)		39 (25.5)		
	Other specialties	130 (19.5)		95 (18.5)		35 (22.9)		
	Both specialties	30 (4.5)		25 (4.9)		5 (3.3)		
	No duty work	365 (54.7)		294 (57.2)		71 (46.4)		
Professional profile		665 (99.7)	2 (0.3)	512 (99.6)	2 (0.4)	153 (100.0)	0 (0.0)	<0.001
	Nurse	187 (28.0)		168 (32.7)		19 (12.4)		
	Resident	92 (13.8)		65 (12.7)		27 (17.7)		
	Medical hospital porters	191 (28.6)		122 (23.7)		69 (45.1)		
	Nursing auxiliary	61 (9.2)		55 (10.7)		6 (3.9)		
	Porter	24 (3.6)		16 (3.1)		8 (5.2)		
	Other	110 (16.5)		86 (16.7)		24 (15.7)		
Age		646 (96.9)	21 (3.1)	496 (96.5)	18 (3.5)	150 (98.0)	3 (2.0)	
Mean (sd)		43.1 (11.8)		42.9 (11.4)		44.1 (13.2)		0.274
Marital status		663 (99.4)	4 (0.6)	510 (99.2)	4 (0.8)	153 (100.0)	0 (0.0)	0.404
	Single	266 (36.9)		208 (40.5)		58 (37.9)		
	Married	357 (53.5)		272 (52.9)		85 (55.6)		
	Divorced	34 (5.1)		24 (4.7)		10 (6.5)		
	Widow/Widower	6 (0.9)		6 (1.2)		0 (0.0)		
History of psychiatric treatment		660 (99.0)	7 (1.00)	509 (99.0)	5 (1.0)	151 (98.7)	2 (1.3)	0.536
	Yes	78 (11.7)		58 (11.3)		20 (13.1)		
	No	582 (87.3)		451 (87.7)		131 (85.6)		
History of COVID-19 diagnosis		665 (99.7)	2 (0.3)	511 (99.4)	3 (0.6)	153 (100.0)	0 (0.0)	0.994
	Yes	65 (9.8)		50 (9.7)		15 (9.8)		
	No	599 (89.8)		461 (89.7)		138 (92.0)		
Having people in charge		667 (100.0)	0 (0.00)	514 (100.0)	0 (0.00)	153 (100.0)	0 (0.0)	0.488
	Yes	339 (50.8)		265 (51.6)		74 (48.4)		
	No	328 (49.2)		249 (48.4)		79 (51.6)		
PSS score		634 (95.1)	33 (4.9)	487 (94.7)	27 (5.3)	147 (96.1)	6 (3.9)	<0.001
Mean (sd)		24.9 (9.6)		25.9 (9.5)		21.6 (9.1)		
GHQ-28 score		667 (100.0)	0 (0.00)	514 (100.0)	0 (0.00)	153 (100.0)	0 (0.0)	
		9.7 (6.5)		10.4 (6.4)		7.1 (6.3)		<0.001

In all job categories, the proportion of women was higher. They made up 90.2 and 89.8% of the total in the Assistant and Nursing category, respectively, differences that reached statistical significance (*p* < 0.001). Regarding caregiver status, proportions were similar (51.6% in woman, 48.4% in men, *p* = 0.536). Eleven point three percent of females reported past psychiatric history, a proportion that was higher in men [(13.1%), *p* < 0.01]. Differences in other variables such as marital and caregiver status, COVID-19 infection, Job Department, or contact with CoVID-19 positive patients did not reach statistical significance (*p* > 0.05).

### GHQ-28 Analysis

The mean score in the GHQ-28 was above the cut-off of 4/5 in women and males (10,44 and 7.10, respectively), but it was significantly higher in women (p < 0.01). Women, widower, hospital porters, COVID-19 infected people, and those who did not make night shifts had the highest scores (eg, mean GHQ-28 scores among widower: 10.0 [6]; mean GHQ-28 scores among hospital porters 14.0 [5,3]; mean GHQ-28 scores among CoVID-19 infected: 11.10 [6.20]; mean GHQ-28 scores among people with psychiatric history 12.50 [8.10]; mean GHQ-28 scores among people without night shifts 10.93 [6.61]). Women doctors (both residents and certified doctors) had significantly higher scores than men (Table 2).

**Table 2:** General Health Questionnary-28 results.

	**GHQ-28**				
	**Females**		**Males**		
**Variable**	**Mean (sd)**	**n**	**Mean (*n*)**	***n***	***p*-value**
Overall	10.4 (6.4)	514	7.1 (6.3)	153	<0.001
**Profesional profile**					
Medical hospital porters	9.2 (6.3)	122	5.0 (4.7)	69	<0.001
Medical residents	9.3 (5.7)	65	5.7 (6.5)	27	0.011
Nurses	11.5 (6.4)	168	9.6 (6.9)	19	0.231
Nursing assistant	11.0 (6.3)	55	6.0 (3.5)	6	0.060
Hospital porters	14.6 (4.2)	16	12.6 (7.0)	8	0.391
Other personnel	9.9 (6.7)	86	10.8 (7.2)	24	0.586
**Night Shifts**					
Medicine specialties	9.7 (6.4)	89	5.4 (5.9)	39	<0.001
Other specialties	8.8 (6.0)	95	5.5 (5.0)	35	0.004
Both specialties	8.6 (5.5)	25	6.0 (3.4)	5	0.312
No duty work	11.4 (6.5)	294	8.9 (6.8)	71	0.005
**Marital status**					
Single	10.1 (6.3)	208	7.9 (6.6)	58	0.023
Married	10.8 (6.5)	272	6.4 (6.3)	85	<0.001
Divorced	9.3 (5.5)	24	7.5 (5.3)	10	0.390
Widow/Widower	10.0 (7.4)	6	–	–	–
**History of psychiatric treatment**					
Yes	13.4 (7.8)	58	9.9 (8.8)	20	0.092
No	10.1 (6.1)	451	6.5 (5.7)	131	<0.001
**History of COVID-19 diagnosis**					
Yes	11.8 (6.3)	50	8.7 (5.5)	15	0.099
No	10.3 (6.4)	461	6.9 (6.4)	138	<0.001
**Having people in charge**					
Yes	10.9 (6.5)	265	6.7 (6.4)	74	<0.001
No	10.0 (6.2)	249	7.4 (6.3)	79	0.001

Case rate was 501 [74.4%]. This proportion was significant higher in women (78.4 vs. 61.3%, *p* < 0.001). Respecting the job category, we observed that all Orderly hospital porters were cases. In women, nurses were the second most affected category, reaching 82.7% of people. This proportion was slightly lower in male nurses, with a total of 78.9% affected. The less affected job category in females was doctors (72.9%), while in the medical resident category, it was men (44.4%).

The multivariant regression analysis showed that orderly hospital porters was associated with significantly higher scores than the rest of the job categories, as did females and people with psychiatric history (**Table 4**). Age inversely influenced the score: each additional year of age was associated with a lower score. We did not find a differential effect of gender combined with job category or being a caregiver. After adjusting for gender as a covariate, only female gender and COVID-19 infection were associated with a higher prevalence of GHQ-28 cases. The risk of being a case was 18% (26-9) inferior by being male, while COVID-19 infection was associated with a 12% (2-21) increase in the risk of a score of 5 or more. (data not shown).

### Perceived Stress Scale Analysis

We found significant differences respecting gender (e.g., mean PSS scores among women vs. males: 10.44 [6.34] vs. 7.06 [6.33], *p* < 0.01). Hospital porters was the job category with higher values [30,1 (7.0)], differences that reached statistical significance respecting other categories (*p* < 0,05). Differences respecting duty work responsibilities, history of psychiatric treatment and history of COVID-19 diagnosis also reached statistically significance. We then compared PSS scores in an intersectional analysis, showed in Table 3.

**Table 3:** Perceived Stress Scale results.

**PSS score**					
	**Females**		**Males**		
**Variable**	**Mean (sd)**	***n***	**Mean (*****n*****)**	**Sd**	***p*****-value**
Overall	25.9 (9.5)	487	21.6 (9.1)	147	<0.001
**Profesional profile**					
Medical hospital porters	23.7 (9.6)	118	19.3 (8.2)	66	0.002
Medical residents	25.7 (9.8)	64	19.7 (10.0)	27	0.010
Nurses	27.2 (9.3)	157	22.8 (8.2)	19	0.052
Nursing assistant	27.7 (8.6)	50	20.0 (6.1)	6	0.039
Hospital porters	30.3 (7.1)	16	29.6 (7.5)	7	0.822
Other personnel	25.01 (9.5)	82	27.7 (8.8)	22	0.239
**Duty work responsibilities**					
Emergency Department	25.0 (9.5)	86	19.8 (10.6)	38	0.008
Other specialties	24.4 (9.4)	91	20.2 (7.1)	33	0.019
Both specialties	24.4 (10.0)	25	18.6 (3.4)	5	0.221
No duty work	26.9 (9.4)	275	23.9 (9.4)	68	0.016
**Marital status**					
Single	25.6 (9.5)	204	22.1 (9.5)	56	0.015
Married	26.4 (9.5)	253	21.0 (8.8)	81	<0.001
Divorced	25.0 (8.7)	22	23.1 (9.7)	10	0.595
Widow/Widower	22.0 (6.3)	4	–	–	–
**History of psychiatric treatment**					
Yes	28.8 (9.2)	56	29.3 (10.6)	19	0.824
No	25.6 (9.4)	428	20.3 (8.2)	127	<0.001
**History of COVID-19 diagnosis**					
Yes	27.3 (9.8)	49	20.7 (8.5)	15	0.023
No	25.8 (9.4)	435	21.7 (9.2)	132	<0.001
**Having people in charge**					
Yes	26.2 (9.5)	243	21.1 (8.8)	70	<0.001
No	25.7 (9.4)	244	22.1 (9.4)	77	0.003

The multivariable regression model showed that job category, female gender, and having previous psychiatric history are associated with worse PSS scores (Table 4). The effect of gender was not influenced by age, marital status, or covid-19 infection. Gender did modify the effect of having a psychiatric history in PSS results, with higher scores in men than in women (21.09 SD: 0.83 vs. 30.11 SD: 2.09).

**Table 4:** Multivariant Regression analysis.

**Characteristic**	**Median GHQ-28 SD**	**Coef**	***p***	**Median PSS**	**Coef**.	***p***
**Gender**						
Female	10.44 (6.34)	Reference		25.94 (9.46)	Reference	
Male	7.06 (6.33)	−3.34 (−4.53, −2.22)	<0.01	21.56 (9.13)	−4.36 (−6.09, −2.62)	<0.01
**Marital status**						
Single	9.64 (6.45)	Reference		24.97 (9.67)	Reference	
Married	9.79 (6.71)	0.15 (−0.89, 1.19)	0.72	25.06 (9.58)	0.09 (−1.45, 1.64)	0.91
Divorced	8.54 (5.51)	−1.10 (−3.41, 1.22)	0.35	24.15 (8.90)	−0.81 (−4.28, 2.66)	0.65
Widower	10 (7.43)	0.36 (−4.95, 5.68)	0.89	22.00 (6.32)	−2.96 (−12.43, 6.50)	0.54
**Technical title**						
Nurse	11.27 (6,49)	Reference		26.70 (9.29)	−3.38 (−7.45, 0.68)	0.10
Medical Resident	8.23 (6.14)	−3.04 (−4.6, −1.45)	<0.01	23.96 (10.20)	−6.13 (−10.41, −1.85)	<0.01
Certified doctor	7.78 (6.16)	−3.49 (−4.77, −2.22)	<0.01	22.11 (9.33)	−7.97 (−12.03, −3.92)	<0.01
Assistant	10.52 (6.21)	−0.75 (−2.58, 1.09)	0.42	26.88 (8.70)	−3.21 (−7.75, 1.33)	0.17
Orderly	13.96 (5.25)	2.68 (−0.01, 5.34)	0.05	30.09 (7.03)	Reference	
Other	10.12 (6.83)	−1.15 (−2.63, 0.33)	0.13	25.73 (9.44)	−4.35 (−8.57, −0.14)	0.04
**Night shift**						
No	10.93 (6.61)	2.47 (1.12, 3.76)	<0.01	26.37 (9.39)	3.01 (1.07, 4.95)	<0.01
Emergency	8.45 (6.63)	Reference		23.36 (10.6)	Reference	
Other department	7.89 (5.89)	−0.56 (−2.12, 1.00)	0.48	23.28 (9.01)	−0.07 (−2.43, 2.27)	0.95
Both	8.20 (5,24)	−0.25 (−2.81, 2.30)	0.84	23.40 (9.48)	0.04 (−3.74, 3.82)	0.98
**Psychiatric history**						
No	9.23 (6.20)	Reference		24.41 (9.44)	Reference	
Yes	12.50 (8.14)	3.20 (1.67, 4.73)	<0.01	28.89 (9.48)	4.48 (2.2, 6.76)	<0.01
**CoVID-19 infection**						
No	9.49 (6.53)	Reference		24.85 (9.54)	Reference	
Yes	11.23 (6.32)	1.73 (0.76, 3.39)	0.04	25.72 (9.89)	0.87 (−1.61, 3.35)	0.49

## Discussion

We performed a gendered analysis of both the quality of health and the perceived stress in the medical workforce during the first wave of the COVID-19 pandemic. Most published studies observed that, within the medical workforce, women had higher levels of stress, anxiety, and depression than males (41–44). However, COVID-19 infected men have worse outcomes than women (56), implying that social determinants of health (like economic aspects, education, and inequality) may explain this difference.

First of all, we observed differences in gender distributions in our sample, as 76.4% of the participants identified themselves as women. The WHO reported that women make 70% of the current medical workforce (37), but this number goes up to 79% in the Basque Health system, where our study took place (38). This proportion is similar to what we found in our study, but selection bias cannot be dismissed as the survey was voluntary and anonymous. If we compare our results with other studies on the impact of the pandemic in occidental countries, gender distributions are similar (9–11, 57). We also found occupational segregation by gender. According to this phenomenon, women in health are clustered in lower status/lower-paid jobs, and at the same time, they occupy jobs culturally labeled as “women's work,” driven by gender stereotypes (58). In our sample, 90.2 and 89.8% of total Nursing Assistants and Nurses, respectively, were women, a proportion that drops to 63.9% in more qualified jobs like physicians. Our data goes in pair with previous WHO reports highlighting the asymmetric distribution of females across healthcare occupations (37). Occupational segregation is a systemic phenomenon in medicine that reaches its maximum gap in leadership positions, where women hold only 25% of senior roles and just 13% of clinical professors in University contracts (58, 59). The importance of this phenomenon lies in the fact that women performed those jobs whose exposure to the virus was higher during the first wave, such as Nurse Assistants and Nurses. Studies later showed that being in the frontline against the virus in direct contact with COVID-19 patients was, in fact, a risk factor for higher psychological distress (24, 60).

We then analyzed the quality of health using the 28-item General Health Questionary and found that the case rate was significantly higher in women than men (78.4 vs. 61.3%, *p* < 0.001). Our data is similar to other studies that report proportions between 45.8 and 80.6% (61–63). These results must be interpreted with caution, as women already have a worse self-reported quality of life, according to the analysis of the World Health Survey of 59 countries (64). However, the case rate in women in the general population lies between 18 and 32.34% (65, 66), a percentage inferior to what we found in our study. Compared with studies on women nurses, (31–36%) (67, 68) and in doctors (27–35%) (69, 70), the proportion is still higher. Therefore, we can conclude that the pandemic could have even doubled the number of healthcare workers at risk of a mental health disorder in our Hospital. More research is needed to quantify the increase in distress in this population.

Similar to other published studies, we found that women had higher mean scores on the PSS scale than men (10.44 [6.34] vs. 7.06 [6.33], *p* < 0.01). The percentage is lower than other studies, though they only provide gender-aggregated data, so we cannot compare our results respecting gender (8, 57, 71, 72). The perceived stress scale measures the level to which an event overcomes the ability of the individual to face stressors, following the Transactional model of stress proposed by Lazarus and Folkman (73). The model emphasizes the person-environment transaction and suggests that individual appraisal processes and coping strategies highly influence a stress response. Previous studies indicated that women scored higher on both total and subscale scores than men (74, 75). The differences could be explained by measurement bias, but many authors concluded that they were actual gender differences (75, 76). Higher levels of perceived stress have been linked to increased cardiovascular, mental, and autoimmune diseases.

One potential explanation for the higher self-perceived stress in women is the new burdens they had to face during the lockdown. Before the pandemic, women doctors were already subject to work-family conflicts (36) that interfered with their career prospects. After the mandatory closening of child care centers and residencies, these duties lied essentially in the informal care of family members. Although both men and women increased the hours spent in household and family duties, the increase was higher in women (28, 31). During the first wave pandemic in our Hospital, many workers were forced to increase their working hours due to the overwhelming number of COVID-19 patients and the lack of personnel, as many became infected. Therefore, women working in direct contact with COVID-19 patients could have had to deal with both an increase in working and household hours. Besides that, new sources of gender discrimination appeared in the medical professions; females were less likely to be tested for the virus (39) and under-represented in authorship in COVID-19 papers (77), but more data is needed to confirm these findings and explore potential causes.

We finally proceeded with an intersectional analysis. We found that married women had worse scores in both scales than married men, something already pointed out in the general population (78). Marriage is, in fact, a protective factor for men and a risk factor for poor mental health in women. Also, women with caretaking duties got significantly higher scores than their men counterparts. One possible explanation is that the amount of time spent in caretaking differs between genders. Women working in the Basque Health system save a mean of 22 h per week to domestic and caretaking chores, compared to 13 h of men, independently of their socio-economic status (38). In the UK, one study found that during the lockdown, women outnumbered men in the weekly hours dedicated to housework, and both men and women had to spend more time in these cores than before (31). There is no data yet on the Spanish population. It would be interesting to study changes during the pandemic, both in the general population and the medical workforce.

Also, in our study, the multivariant regression analysis showed that previous psychiatric history is a risk factor for higher perceived stress, and this effect is significantly higher in men. Following our results, data from the MIND/COVID research group also point out that psychiatric history is a risk factor for distress in the medical population (24). In another publication, they analyzed suicidal behavior during the first wave in the medical workforce and found that being men is a risk factor for suicide thoughts and committed suicide (25). Due to gender socialization, men's identity is shaped under a masculine stereotype in which emotions are repressed, and the expression of feelings and needs are seen as weaknesses (79). Conforming to the masculine role can be deleterious to men's mental health and is related to the increased rate of suicides in men (80). We presume that during a particularly stressful situation like the pandemic, men working in healthcare could have repressed their emotions and avoided asking for help, following the masculine gender role. This fact could be aggravated in those with a previous psychiatric history. However, more research is needed to confirm whether male medical professionals who have psychiatric diseases constitute a risk group.

### Strengths and Limitations

One of the limitations of our study is the sampling method used: we used convenience sampling, so our sample was formed by those subjects that voluntarily completed the online survey. We chose this method for several reasons: first of all, our study took place during the first wave of the pandemic, when there was an urgent need for pilot data of the psychological impact of the pandemic, and convenience sampling was the most cost-effective and speedy method of recruitment. Due to the risk of infection and virus transmission, it was not possible to conduct face-to-face interviews, so an online questionary permitted a safe way of evaluation. Another limitation of this study is that only those hospital workers with institutional email addresses were eligible, which in our health systems excludes food, cleaning, and ambulance services. Therefore, these collectives were not included in the analysis.

The strengths of our analysis lie in the gender analysis and the provision of gender-disaggregated data. According to the European Union research recommendations, gender analysis should be incorporated in research, and they even provide guidelines to ease its incorporation by researchers (81). However, most published works barely mention gender and neither provide gender-disaggregated data. Also, our study took place during the first wave of the pandemic, when there was a mandatory lookdown in Spain. It will be of particular interest to compare these results with future data and check the evolution of the mental health of the medical workforce.

## Conclusions

The coronavirus pandemic has meant an enormous psychological burden for the medical workforce. Medical professionals, especially women, experienced high levels of distress and a worse quality of life during the pandemic, and they are at high risk of mental health disorders. For this reason, there should be psychological interventions to aid healthcare workers coping with the coronavirus pandemic, and they should be designed from the gender perspective. The Basque Country Health System (38), recognizes that women in healthcare have to cope with strains such as economic inequalities, under-recognition, a higher ratio of household chores, and increased children care expectations. Public recommendations and policies should not be general, ignoring the reality of gender bias and, as other authors claim (82), governments and global health institutions should consider the sex and gender effects of the COVID-19 outbreak, both direct and indirect, and develop gender-sensitive policies.

## Data Availability Statement

The raw data supporting the conclusions of this article will be made available by the authors, without undue reservation.

## Author Contributions

ML-A, JG-B, EL-G, and RS designed the study. ML-A, JG-B, EL-G, and MR-B collected the data. JP-Z, MS-H, ML-A, and JG-B analyzed the data. ML-A, JG-B, JP-Z, AG-P, and MS-H wrote the article. All authors approved the final work.

## Conflict of Interest

The authors declare that the research was conducted in the absence of any commercial or financial relationships that could be construed as a potential conflict of interest.
